# Arginine glycosylation regulates UDP-GlcNAc biosynthesis in *Salmonella enterica*

**DOI:** 10.1038/s41598-022-09276-9

**Published:** 2022-03-28

**Authors:** Samir El Qaidi, Nichollas E. Scott, Michael P. Hays, Philip R. Hardwidge

**Affiliations:** 1 College of Veterinary Medicine, Kansas State University, Manhattan, KS 66506 USA; 2 Department of Microbiology and Immunology, University of Melbourne Within the Peter Doherty Institute for Infection and Immunity, Melbourne, 3000 Australia

**Keywords:** Biochemistry, Microbiology

## Abstract

The *Salmonella enterica* SseK1 protein is a type three secretion system effector that glycosylates host proteins during infection on specific arginine residues with *N*-acetyl glucosamine (GlcNAc). SseK1 also Arg-glycosylates endogenous bacterial proteins and we thus hypothesized that SseK1 activities might be integrated with regulating the intrabacterial abundance of UPD-GlcNAc, the sugar-nucleotide donor used by this effector. After searching for new SseK1 substrates, we found that SseK1 glycosylates arginine residues in the dual repressor-activator protein NagC, leading to increased DNA-binding affinity and enhanced expression of the NagC-regulated genes *glmU* and *glmS*. SseK1 also glycosylates arginine residues in GlmR, a protein that enhances GlmS activity. This Arg-glycosylation improves the ability of GlmR to enhance GlmS activity. We also discovered that NagC is a direct activator of *glmR* expression. *Salmonella* lacking SseK1 produce significantly reduced amounts of UDP-GlcNAc as compared with *Salmonella* expressing SseK1. Overall, we conclude that SseK1 up-regulates UDP-GlcNAc synthesis both by enhancing the DNA-binding activity of NagC and by increasing GlmS activity through GlmR glycosylation. Such regulatory activities may have evolved to maintain sufficient levels of UDP-GlcNAc for both bacterial cell wall precursors and for SseK1 to modify other bacterial and host targets in response to environmental changes and during infection.

## Introduction

The Gram-negative bacterium *Salmonella enterica* is a human and animal pathogen and one of the most common causative agents of food-borne diseases^1,2^. This pathogen acquired two pathogenicity islands that each encode a type III secretion system (T3SS) apparatus and numerous effector proteins that subvert host cell functions^3–5^. The SseK enzymes are T3SS effector glycosyltransferases that glycosylate target proteins on arginine residues^6,7^. Many *Salmonella enterica* genomes encode up to three SseK paralogs, SseK1, SseK2, and SseK3. The NleB enzymes from *E. coli* and *Citrobacter rodentium* are SseK orthologs. At a structural level, these enzymes are composed of three major domains, a catalytic domain that includes DXD and HEN motifs, a helix-loop-helix (HLH) domain, and a C-terminal lid domain^8–10^. These enzymes are important to bacterial virulence because they disrupt host innate immune signaling pathways by Arg-glycosylating multiple proteins, including the FAS-associated death domain-containing protein (FADD) and the tumor necrosis factor receptor (TNFR) type 1-associated death domain protein (TRADD)^6,7^. Glyceraldehyde-3-phosphate dehydrogenase (GAPDH), the transcriptional regulator of cellular O_2_ homeostasis Hif1α, and the tubulin-binding cofactor B (TBCB), are also glycosylation targets of some of the NleB/SseK orthologs^11–14^. Subcellular fractionation experiments facilitated the identification of the Rab GTPases Rab1, Rab5, and Rab11 as targets of SseK3^15^. NleB2 is a bacterial Arg-glucose transferase that glucosylates RIPK1^16^.

We and others have demonstrated that NleB/SseK not only glycosylate host cell proteins but also modify bacterial proteins to improve bacterial survival under hostile environmental conditions. For example, *C. rodentium* NleB Arg-glycosylates the glutathione synthase GshB, leading to enhanced glutathione synthase activity and consequently increased resistance to oxidative stress^17^. SseK1 Arg-glycosylates and enhances the activity of several enzymes (GloA, GloB, GloC, and YajL) involved in methylglyoxal detoxification^18^. It was recently described that SseK3-mediated Arg-glycosylation also plays an important role in modulating the DNA-binding activity of *Salmonella* PhoP, suggesting a mechanism for how Arg-glycosylation may also act as a regulator of gene transcription^19^.

UDP-GlcNAc is an essential precursor for cell wall biosynthesis and the nucleotide-sugar donor for SseK glycosyltransferase activity. UDP-GlcNAc synthesis is mediated through four steps catalyzed by glucosamine-6-phosphate synthase (GlmS), phosphoglucosamine mutase (GlmM), and the bi-functional enzyme glucosamine-1-phosphate acetyltransferase/*N*-acetylglucosamine-1-phosphate uridyltransferase (GlmU)^20^. UDP-GlcNAc synthesis has multiple levels of regulation, including transcriptional activation of the *glmUS* operon by the NagC dual regulator^21^, as well as post-transcriptional regulation of *glmS* mRNA levels through the action of the small RNAs GlmY and GlmZ^22–24^. In *Bacillus subtilis*, another level of GlmS regulation involves the interaction of the UDP-GlcNAc binding protein GlmR with GlmS. GlmR binding to GlmS enhances the glucosamine-6-phosphate synthase activity of GlmS^25,26^.

This study was performed to investigate whether UDP-GlcNAc biosynthesis is regulated by the SseK paralogs in *Salmonella*. Here we report that SseK1 Arg-glycosylates NagC. As a result of this glycosylation, NagC affinity to its target promoters is increased, leading to greater downregulation of the *nagE-BACD* operon encoding the proteins required for GlcNAc uptake and metabolism^27^, as well as greater activation of *glm*US operon. We also identified *glmR*, which encodes the GlmS enhancer, GlmR (also known as YvcK) as a NagC-activated gene. GlmR is also Arg-glycosylated by SseK1 and this glycosylation improves its GlmS enhancer activity, leading to increased glucosamine-6-phosphate synthesis by GlmS. Consequently, SseK1-deficient bacteria are impaired for UDP-GlcNAc production. Thus, SseK1 regulates two aspects of UDP-GlcNAc regulation via Arg-glycosylation to maintain bacterial UDP-GlcNAc homeostasis. Combined, these results support a growing body of evidence that bacterial Arg-glycosylation plays additional regulatory roles in fine-tuning pathogen activities.

## Results

### SseK1 glycosylates NagC and GlmR

**Figure 1:**
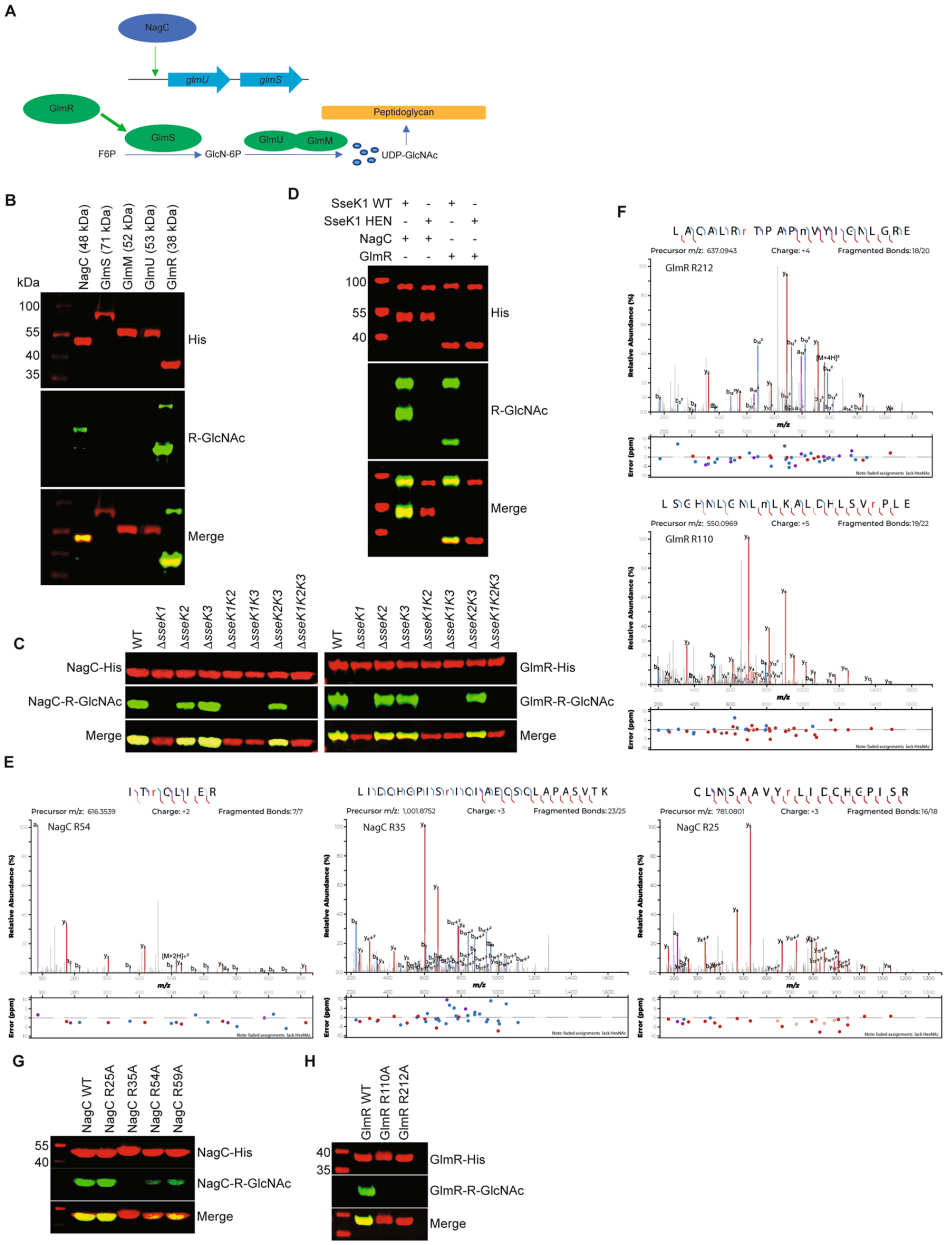
SseK1 glycosylates NagC and GlmR. (**A**) Schematic of NagC and Glm pathway under investigation. (**B**) Western blot analysis of intra-bacterial glycosylation of NagC, GlmS, GlmM, GlmU, and GlmR. (**C**) In vivo glycosylation of indicated proteins in wild-type *Salmonella enterica* and *sseK* mutants. (**D**). Western blot analysis of in vitro glycosylation of NagC and GlmR in the presence of active or inactive (HEN) forms of SseK1. (**E**) Mass spectrometry analysis of NagC Arg-glycosylation by SseK1. HCD spectra of the in vivo glycosylated NagC tryptic peptides containing glycosylated R^54^, R^35^, and R^25^. (**F**) Mass spectrometry analysis of GlmR Arg-glycosylation by SseK1. HCD spectra of the in vivo glycosylated GlmR GluC derived peptides containing glycosylated R^212^, and R^110^. (**G**) Western blot verification of Arg-glycosylation of WT and R-to-A point mutations of NagC. (**H**) Western blot verification of Arg-glycosylation of WT and R-to-A point mutations of GlmR.

Because UDP-GlcNAc is both an essential precursor of the bacterial cell wall and the nucleotide-sugar donor for SseK1, we hypothesized that consumption of UDP-GlcNAc by SseK1 might be compensated by a regulatory mechanism involving SseK1 itself. To test this hypothesis, we first determined whether SseK1 glycosylates any of the proteins involved in UDP-GlcNAc biosynthesis and regulation, namely GlmM, GlmR, GlmS, GlmU, and NagC (Fig. 1A). We expressed His-tagged forms of these proteins in wild-type *Salmonella* and used Western blotting to detect their potential Arg-glycosylation. We found that NagC and GlmR, but not GlmS, GlmM, or GlmU were Arg-glycosylated in wild-type (WT) *Salmonella* (Fig. 1B). To determine the role of SseK enzymes in this phenotype, we evaluated Arg-glycosylation of NagC and GlmR in all potential combinations of mutants possessing or lacking the SseK1, SseK2, and SseK3 enzymes. We found that Arg-glycosylation of NagC and GlmR was completely dependent upon SseK1 (Fig. 1C). We corroborated these data using in vitro glycosylation reactions and found that NagC and GlmR were Arg-glycosylated by WT SseK1, but not by an inactive form of SseK1 (HEN mutant) (Fig. 1D).

To further corroborate these Western blotting data and to determine on which arginine residues the glycosylation occurred, we subjected NagC and GlmR to mass spectrometry analysis. Data from these experiments indicated that NagC is glycosylated on R25, R35, and R54 (Fig. 1E), and GlmR is glycosylated on R110 and R212 (Fig. 1F). While our initial mass spectrometry analysis indicated potential NagC R59 glycosylation, manual inspection of the MS data revealed it was the R54 site that was modified. However, to be comprehensive in our in vitro and in vivo studies, we still elected to mutate this site for analysis.

We used site-directed mutagenesis to corroborate the mass spectrometry data and found that mutating R35 to alanine abolished NagC Arg-glycosylation, whereas mutating R54 and R59 partially reduced NagC Arg-glycosylation, and mutating R25 had no impact on NagC Arg-glycosylation (Fig. 1G). These experiments were performed in vivo within wild-type Salmonella that expressed each of the indicated NagC mutants. These data suggest that R35, R54, and R59 are the primary NagC residues glycosylated by SseK1. We also found that mutating either R110 or R212 abolished GlmR Arg-glycosylation, suggesting that both R110 and R212 are essential for SseK1-mediated Arg-glycosylation (Fig. 1H).

### SseK1-mediated glycosylation of NagC enhances DNA binding

NagC is a transcription factor that coordinates amino-sugar metabolism in bacteria. NagC is a repressor of the *nagE-BACD* divergent operon involved in GlcNAc uptake and metabolism^28^ and is an activator of the *glmUS* operon which encodes the GlmU and GlmS enzymes required for UDP-GlcNAc synthesis^21^. In the absence of GlcNAc, the divergent *nagE-BACD* operon is repressed and the *glmUS* operon is activated. GlcNAc is transported and phosphorylated by a GlcNAc-specific phosphotransferase transporter encoded by NagE. The product GlcNAc-6P, a NagC-regulon inducer, binds to NagC and interferes with its DNA binding activity, leading to de-repression of NagC-repressed genes (*nagE-BACD* operon, for example) and de-activation of NagC-activated genes (*glmUS* operon)^27,29^.

**Figure 2:**
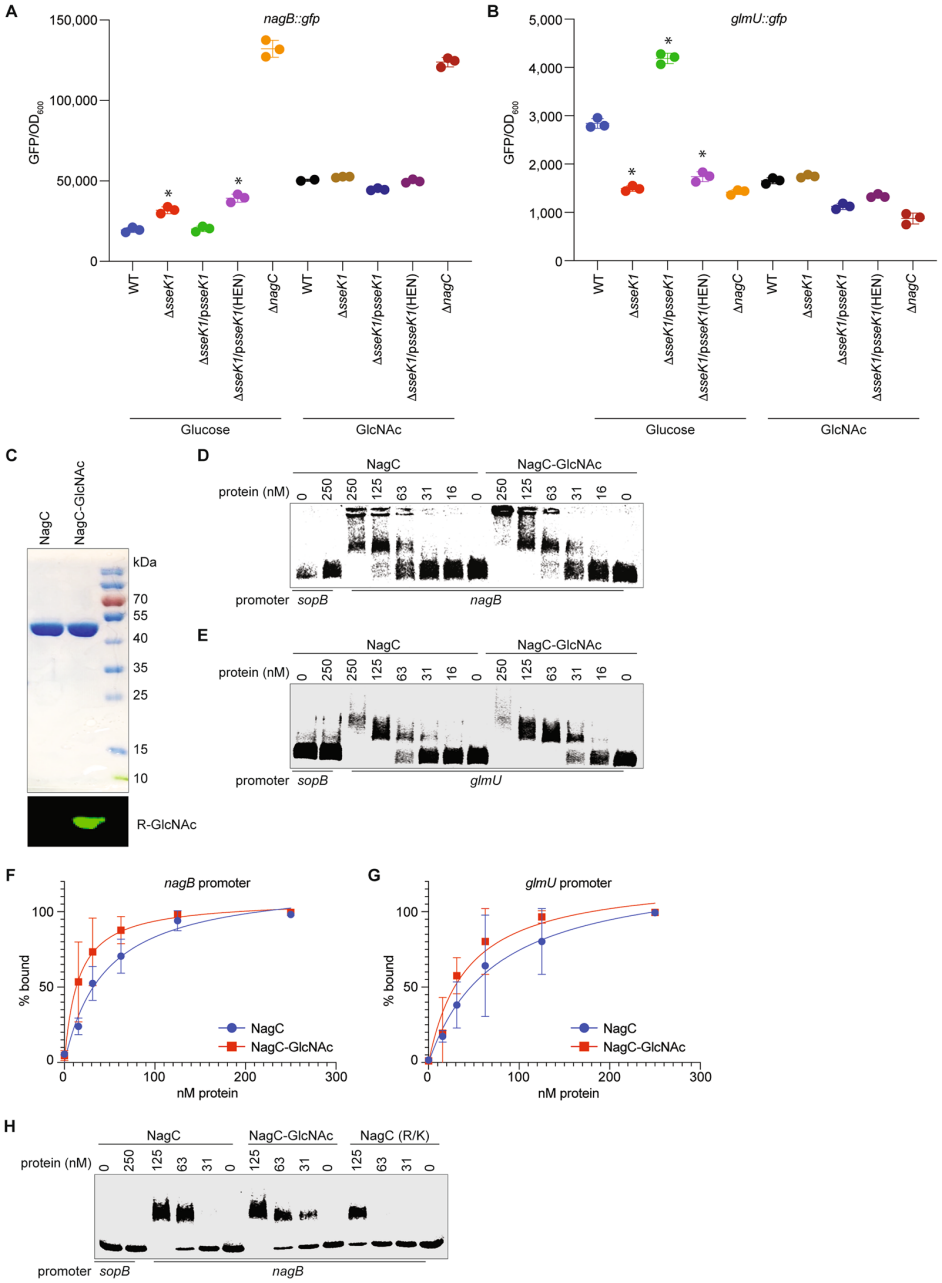
Arg-glycosylation of NagC enhances its DNA binding activity*. *(**A**) Quantification of GFP expression levels of *nagB::gfp* transcriptional fusions in wild-type *Salmonella enterica* and its Δ*sseK1* or Δ*nagC* derivatives grown in M9 minimal medium supplemented with either 0.2% glucose or 0.2% GlcNAc as the sole carbon source for 8 h. GFP levels are expressed as RFU (relative fluorescence units)/OD_600_ ratio. (**B**) Quantification of *glmU::gfp* transcriptional fusions. (**C**) SDS-PAGE and Western blot analysis of purified NagC and NagC-GlcNAc. (**C**) Purification of native and Arg-glycosylated NagC combined with validation of glycosylation by using Western blotting. (**D**) EMSAs comparing the DNA-binding activity of NagC and NagC-GlcNAc towards *nagB* promoter DNA. Two nmoles of Alexa fluor-labeled and then DNA–protein complexes were resolved on 0.5% agarose gels. (**E**) *glmU* EMSAs. (**F**) Quantification of binding affinities of native and Arg-glycosylated NagC to *nagB* promoter DNA. (**G**) Quantification of binding affinities of native and Arg-glycosylated NagC to *glmU* promoter DNA. (**H**) EMSAs comparing the DNA-binding activity of NagC, NagC-GlcNAc, and NagC (R–K) mutant towards *nagB* promoter DNA. Experiments were performed as described for panel D.

To determine the significance of SseK1-mediated glycosylation of NagC in vivo, we constructed transcriptional fusions of either the *nagB* promoter, which is repressed by NagC, or the *glmU* promoter, which is activated by NagC, to the green fluorescence protein (GFP). We measured GFP levels in wild-type (WT) *Salmonella* and in Δ*sseK1* backgrounds after bacteria were grown in M9 minimal medium supplemented or not with GlcNAc. No differences in bacterial growth rates among strains were observed. In the absence of GlcNAc, repression of the *nagB-gfp* fusion was more pronounced in WT *Salmonella* than in Δ*sseK1 Salmonella* (Fig. 2A). Complementation of Δ*sseK1* with an active form of *sseK1* but not an inactive form of *sseK1*[*sseK1*(HEN)] restored GFP expression to levels observed for the WT strain. The Δ*nagC* mutant was used as a positive control for these assays. Conversely, greater *glmU-gfp* expression was seen in WT, as compared to Δ*sseK1* (Fig. 2B) and complementation of the Δ*sseK1* mutant restored the expected phenotypes. Addition of GlcNAc to the medium rendered the GFP expression levels insensitive to SseK1, since a similar level of *nagB-gfp* de-repression and *glmU-gfp* de-activation was observed in both WT and Δ*sseK1* strains. Note that we used M9 minimal medium for these experiments specifically so that we could study the impact of SseK1 on *nag* and *glm* operon expression. Others have shown that significant amounts of SseK1 are produced in both LB and in LPM minimal medium^30^. We determined by using both RT-PCR and Western blotting that, in M9 minimal medium, SseK1 is expressed and is active (data not shown).

The increased repression of the *nagB-gfp* fusion and the higher activation of the *glmU-gfp* fusion in WT *Salmonella* in the absence of GlcNAc might be explained if SseK1-mediated Arg-glycosylation of NagC increases NagC affinity to its cognate promoters. To test this hypothesis, native and Arg-glycosylated forms of NagC were purified after their co-expression in *E. coli* BL21(DE3) cells (Fig. 2C) and their DNA-binding affinities were quantified by using Electrophoretic Mobility Shift Assays (EMSAs). Alexa fluor-labeled DNA duplexes corresponding to either the *nagB* or *glmU* promoters were incubated with NagC and complexes were resolved on agarose gels. The *sopB* promoter was used as a negative control to assess any non-specific DNA binding by NagC. Consistent with the transcriptional fusion data, Arg-glycosylated NagC-GlcNAc showed ~ 2 to threefold stronger affinity to the *nagB* and *glmU* promoters, as compared to the unglycosylated form of NagC (Figs. 2D, E). Unglycosylated NagC bound with 45.3 nM affinity to the *nagB* promoter; Arg-glycosylation of NagC improved the affinity to 15.1 nM (Fig. 2F). Unglycosylated NagC bound with 74 nM affinity to the *glmU* promoter; Arg-glycosylation of NagC improved the affinity to 42 nM (Fig. 2G). To determine the role of basic character of the amino acids targeted by SseK1 in mediating affinity to the *nagB* promoter, we generated and purified a NagC mutant in which we converted the R25, R35, R54, and R59 residues to lysines. We found that this NagC (R-K) mutant had significantly reduced affinity for the nagB promoter (Fig. 2H).

### NagC activates glmR expression

**Figure 3:**
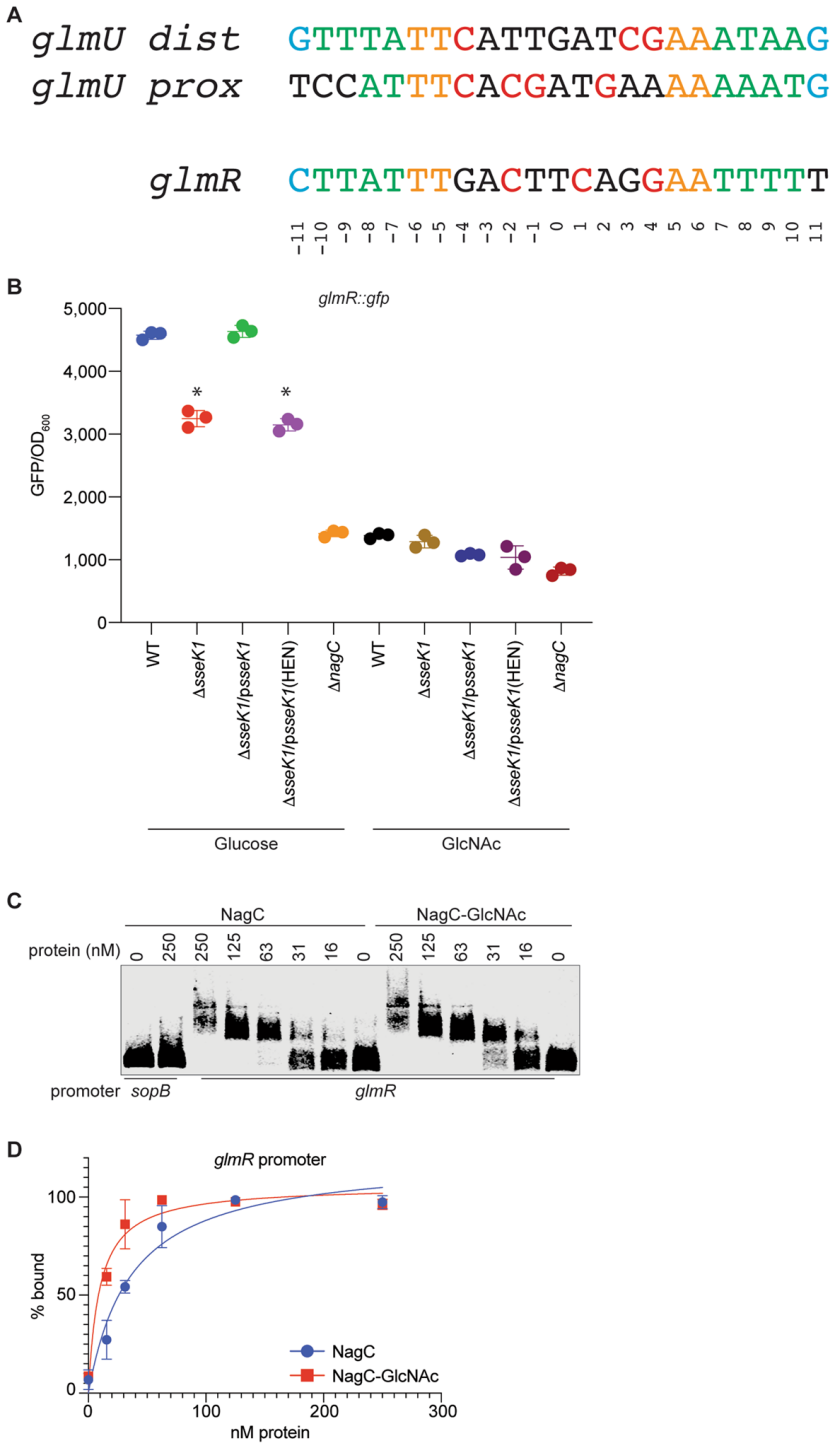
NagC activates *glmR*. (**A**) Identification of a potential NagC binding site in the *glmR* promoter based on similarity to the *glmU* promoter. (**B**) Quantification of GFP expression levels of *glmR*::*gfp* transcriptional fusions in wild-type *Salmonella enterica* and its Δ*sseK1* or Δ*nagC* derivatives grown in M9 minimal medium supplemented with either 0.2% glucose or 0.2% GlcNAc as the sole carbon source for 8 h. GFP levels are expressed as RFU (relative fluorescence units)/ OD_600_ ratio. (**C**) EMSAs comparing the DNA-binding activity of NagC and NagC-GlcNAc towards *glmR* promoter DNA. (**D**) Quantification of binding affinities.

As compared with *glmU*, less is known regarding *glmR* regulation. Because this gene encodes a UDP-GlcNAc binding protein that is important for GlmS activity^26^, we hypothesized that this gene might be regulated by NagC. We found in the *glmR* promoter region a DNA motif that is similar to the cognate NagC-binding site in *glmU*, consisting of a 23 base pair pseudo-palindrome with a GC-rich central region flanked by the characteristic T/T and A/A motifs at the −11/−10 and +10/+11 positions respectively, as well as an external AT-rich region (Fig. 3A). To determine whether NagC regulates *glmR* expression, we constructed a *glmR-gfp* fusion and measured GFP expression in WT *Salmonella* in the presence or absence of GlcNAc (NagC inducer). We found that reduced *glmR-gfp* expression was seen in the Δ*sseK1* mutant as compared to WT *Salmonella*. *glmR-gfp* expression levels were increased in the absence of GlcNAc, suggesting that NagC is an activator of *glmR* (Fig. 3B). Consistent with the other EMSAs (Fig. 2) and these transcriptional fusion data, Arg-glycosylated NagC-GlcNAc showed ~ threefold stronger affinity to the *glmR* promoter, as compared to the unglycosylated form of NagC (Fig. 3C). Unglycosylated NagC bound with 36 nM affinity to the *glmR* promoter; Arg-glycosylation of NagC improved the affinity to 10 nM (Fig. 3D). The *sopB* promoter was again used as a negative control to assess any non-specific DNA binding by NagC.

### GlmR Arg-glycosylation enhances its GlmS enhancer activity

**Figure 4:**
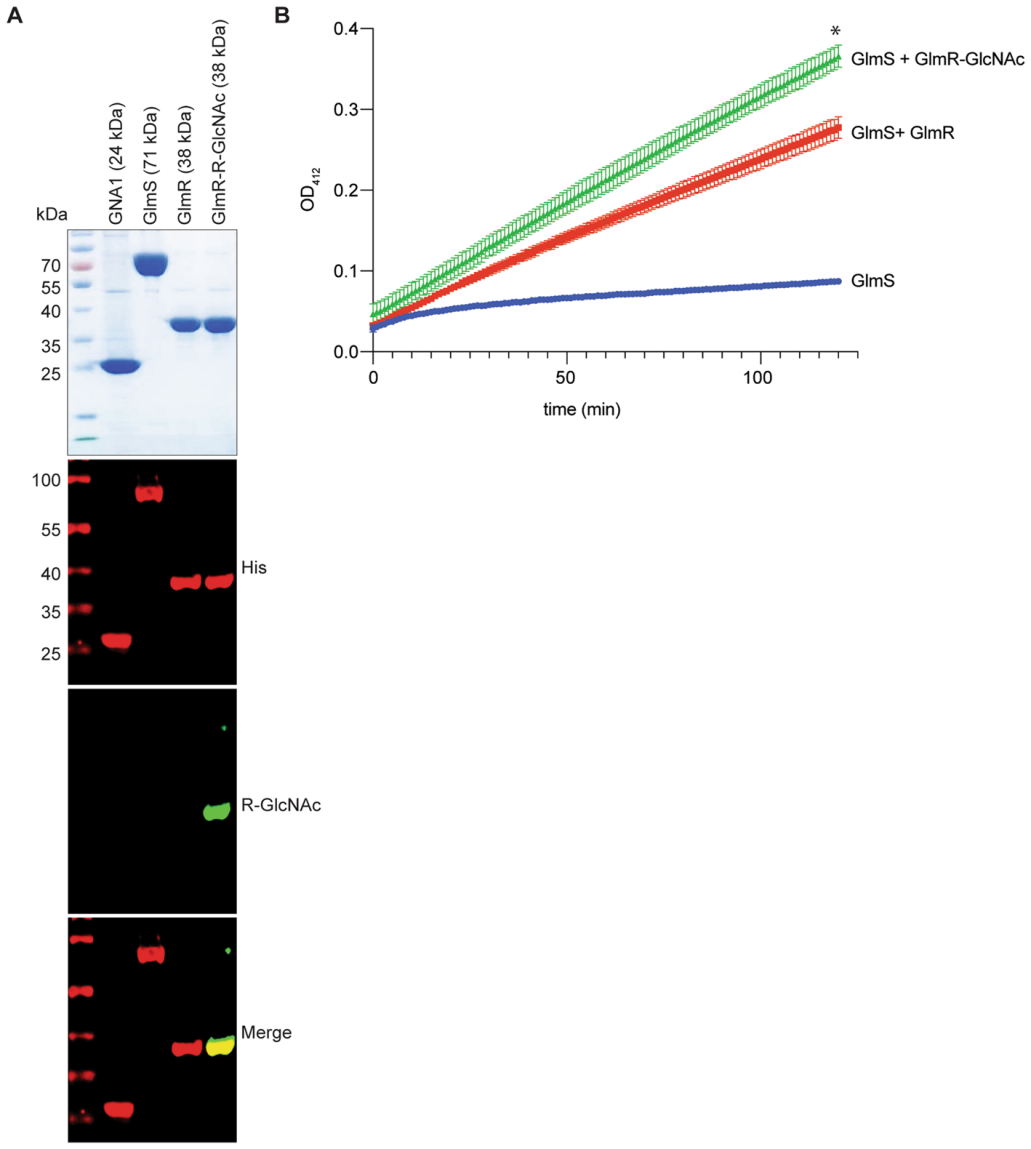
Arg-glycosylation of GlmR improves its GlmS enhancer activity. (**A**) SDS-PAGE and Western blot analysis of purified proteins used for GlmS activity assays. (**B**) GlmS activity was measured in the presence of absence of either native or Arg-glycosylated GlmR by measuring the amount of CoASH produced in an enzyme-coupled assay. The GlmS product GlcN6P was acetylated by the yeast GlcN6P *N*-acetyltransferase 1, GNA-1, to produce GlcNAc6P and CoASH which was measured at 412 nm.

In *Bacillus subtilis*, GlmR interacts with GlmS when UDP-GlcNAc concentrations are low^26^. This interaction is crucial to enhancing the D-fructose-6-phosphate aminotransferase activity of GlmS^26^. Since we identified GlmR as an SseK1 glycosylation target, we next assessed the impact of GlmR glycosylation on GlmS activity. To measure GlmS activity, we conducted an assay in which the GlmS product GlcN6P is acetylated by the yeast GlcN6P *N*-acetyltransferase 1, GNA-1, to produce GlcNAc6P and CoASH^31^. GlmS, GNA-1, GlmR and GlmR-GlcNAc were purified and the Arg-glycosylation of purified GlmR-GlcNAc was confirmed by using Western blotting (Fig. 4A). We observed that the Arg-glycosylated form of GlmR significantly increased GlmS activity, as compared to the unglycosylated form of GlmR (Fig. 4B).

### UDP-GlcNAc levels are higher in WT than ΔsseK1 Salmonella

**Figure 5:**
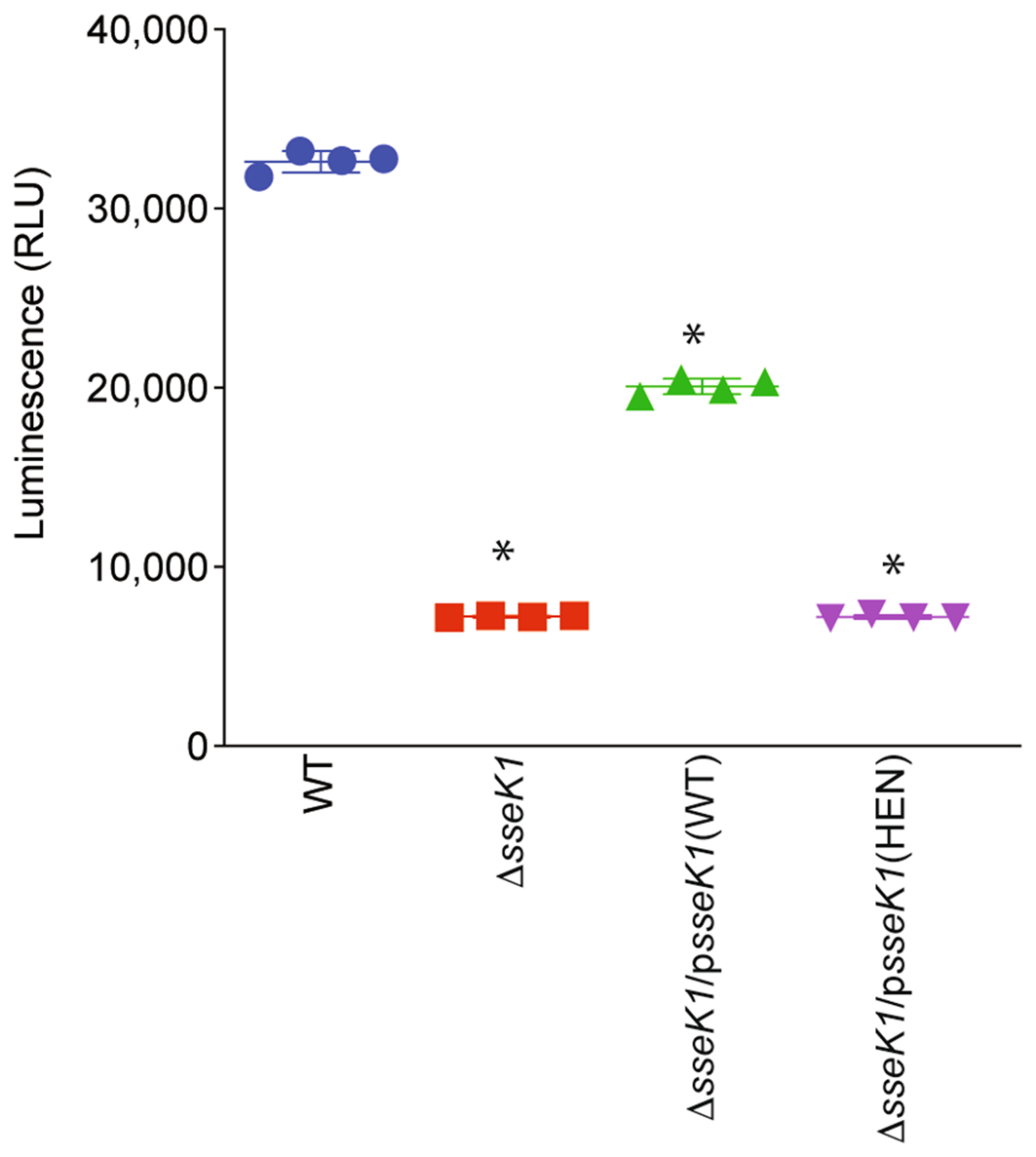
Quantification of UDP-GlcNAc levels*.* Wild-type *Salmonella,* Δ*sseK1*, and complemented Δ*sseK1* strains were grown overnight in M9 medium. Cell lysates were incubated for 2 h at room temperature with 100 mM SseK1 to hydrolyze UDP-GlcNAc. The UDP was quantified by using a UDP detection reagent (Promega) that converts UDP into ATP to generate light in a luciferase reaction.

To evaluate the consequence of NagC and GlmR Arg-glycosylation by SseK1 on UDP-GlcNAc levels in *Salmonella*, we measured the UDP-GlcNAc levels in WT and the Δ*sseK1* mutant. Cell lysates from WT or Δ*sseK1 Salmonella* were incubated in vitro with SseK1 to hydrolyze UDP-GlcNAc into UDP and GlcNAc. The generated UDP was then converted into ATP for use in luciferase assays (Fig. 5). WT *Salmonella* produced significantly higher amounts of UDP-GlcNAc than the Δ*sseK1* mutant. WT levels of UDP-GlcNAc were partially restored upon complementation with an active form of SseK1 but not with the inactive HEN mutant.

## Discussion

**Figure 6:**
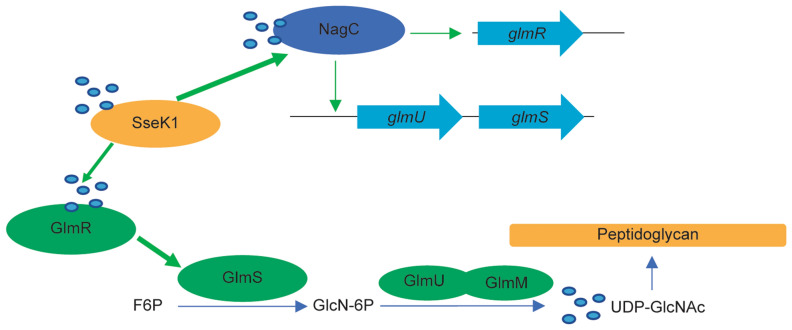
Working Model. SseK1 Arg-glycosylates NagC to enhance transcriptional activation of *glmUS* and *glmR.* SseK1 also glycosylates GlmR to increase GlmS activity, leading to increased levels of UDP-GlcNAc.

SseK1 is a T3SS effector that glycosylates target proteins with GlcNAc on arginine residues. Within the host, SseK1-mediated glycosylation of target proteins interferes with the proper function of adaptor proteins in signaling pathways, leading to reduced host inflammatory response against the pathogen. Within the bacterium, SseK1-mediated glycosylation of target proteins leads to, in addition to the phenotypes we show here, enhanced resistance to methylglyoxal^18^. Here we found that to promote UDP-GlcNAc production, SseK1 Arg-glycosylates two proteins that regulate different aspects of UDP-GlcNAc biosynthesis (Fig. 6). First, by enhancing the ability of NagC to regulate the *glmUS* operon and the *glmR* gene; second, by improving the ability of GlmR to enhance GlmS activity. This regulatory mechanism may allow *Salmonella* to maintain sufficient levels of UDP-GlcNAc for cell well synthesis and for the glycosylation of other bacterial and host proteins (Fig. 6).

SseK1 glycosylates arginine residues that are in or near the NagC HTH domain that is responsible for DNA binding, suggesting that the significance of targeting NagC by SseK1 is to modulate its DNA binding affinity towards target gene promoters. Upon Arg-glycosylation, NagC bound with higher affinity to the *nagB*, *glmU*, and *glmR* promoters. Since NagC is a pleiotropic regulator that controls the expression of multiple genes involved in several metabolic pathways, its glycosylation by SseK1 adds an additional layer of gene regulation to this pathogen for the fine-tuning of gene expression in response to bacterial needs.

Several pathogens have evolved mechanisms to modulate GlmR activity. For example, in *Bacillus subtilis,* GlmR interacts with either GlmS or YvcJ depending on UDP-GlcNAc availability^26^. In the presence of non-glycolytic carbon sources such as intermediates of the Krebs cycle, GlmR binds to GlmS and enhances its catalytic activity. In the presence of glycolytic carbon sources such as glucose, UDP-GlcNAc concentrations are sufficient and GlmR instead binds to YvcJ to avoid excessive stimulation of GlmS and unnecessary production of UDP-GlcNAc^26^. In *Listeria monocytogenes*, GlmR (YcvK) is phosphorylated by the serine protein kinase PkrA; regulation of GlmR phosphorylation is critical for virulence and cytosolic survival^32^. In this study, we found that in *Salmonella enterica*, which lacks YcvJ or PrkA homologs, SseK1 glycosylates GlmR to enhance GlmS activity. Future analysis of how GlmR Arg-glycosylation affects its affinity for GlmS may provide a mechanistic explanation for this observation. We also desire in the future to conduct experiments to assess the impact of GlmR glycosylation and/or mutation on *Salmonella* virulence. We have not yet performed any specific experiments to assess cell-wall related phenotypes in the *sseK1* mutant, some of which might be postulated by the differential levels of UDP-GlcNAc we observed. However, we note that the growth rates between the WT and the *sseK1* mutant were not significantly different (*data not shown*), suggesting that there is not a globally-significant impact to the bacterial cell wall due to SseK1 activity, at least under these specific experimental conditions.

O-linked glycosylation of eukaryotic transcription factors is relatively common and has been studied for decades^33^. Such glycosylation can both increase or decrease gene expression^33^. For example, the MORC family CW-type zinc finger 2 protein (MORC2), a chromatin-remodeling enzyme involved in DNA-damage response, exhibits higher transcription activation activity upon O-GlcNAc transferase (OGT)-mediated glycosylation at T556^34^. Another study describes a direct correlation between glycosylation of the Hedgehog pathway transcription factors GLI1 and GLI2 and their transcriptional activity^35^.The pancreatic/duodenal homeobox-1 protein (PDX1), which is required for pancreatic function and development is glycosylated by OGT at high glucose concentrations, leading to increased DNA-binding affinity and consequently greater insulin secretion^36^. OGT also glycosylates the NF-κB c-Rel subunit on S350, a process required for c-Rel DNA binding and transactivation functions^37^. However, in most cases, transcription factor glycosylation also affects their nuclear translocation, and there are relatively few direct measurements of the impact of transcription factor glycosylation on affinity for target gene promoters. One the few examples where both nuclear localization and DNA affinity of a glycosylated transcription factor were assessed is illustrated by the impact on the NF-κB p65 subunit by OGT-mediated glycosylation, leading to aggravated TNF-α-stimulated inflammation both in vitro and in vivo^38^.

By contrast, our study links Arg-glycosylation directly to differential DNA-binding affinity. We observed that Arg-glycosylated NagC bound with higher affinity to the *nagB*, *glmU*, and *glmR* promoters than did the unglycosylated form of NagC. To some extent, these results are counter-intuitive, because one might reasonably expect that glycosylating a basic amino acid would tend to reduce protein affinity for DNA. We also note that, in a related system, the recent analysis of SseK3 glycosylation of PhoP concluded that there was a slight reduction in PhoP affinity for DNA as a function of PhoP R215 glycosylation, although the binding affinities were not calculated^19^. However, it is not clear from these studies whether the investigators used phosphorylated PhoP or recombinant, non-phosphorylated PhoP for their EMSAs, a variable which might affect the interpretation of these data.

The work described here provides the first evidence that Arg-glycosylation of the bacterial transcription factor NagC by SseK1 increases NagC affinity for DNA. These data also represent, to our knowledge, along with the recently described work regarding Arg-glycosylation of PhoP by SseK3^19^, the first example of a T3SS effector directly regulating gene expression by modifying a transcription factor. Future experiments aiming to solve the co-crystal structure of an Arg-GlcNac-NagC-DNA complex may permit a molecular understanding of how Arg-glycosylation of transcription factors alters their interactions with DNA.

## Materials and methods

### Plasmids, strains, and cloning

The plasmids and strains used in this study are listed in Tables 1 and 2, respectively. Wild type *sseK1* (*Salmonella enterica*) and its derivative H244A E255A N256A, were cloned into pET42a. *nagC, glmR, glmU, glmS and glmM* were cloned in p*Tac* using ABC cloning^40^. *nagC* deletions were constructed using lambda red recombination with the pKD3 and pKD119 plasmids^41^. Mutants were screened on LB medium supplemented with 10 µg/ml chloramphenicol and mutations were confirmed by PCR and DNA sequencing. Protein purification was performed as described previously^12^. For the purification of glycosylated substrates, His-tagged substrates of SseK1 were co-expressed (or not) with FLAG-tagged SseK1 and purified against the His-epitope, as described previously^18^.

**Table 1:** Plasmids used in this study.

Construct	Plasmid	Source
**Recombinant proteins**		
Flag-SseK1	pFLAG-CTC-*sseK1*	^10^
Flag-SseK1 (HEN)	pFLAG-CTC-*sseK1* H244A E255A N256A	^10^
GST-SseK1	pET42a-*sseK1*	^11^
GST-SseK1 (HEN)	pET42a-*sseK1* H244A E255A N256A	^10^
His-NagC	*pTac-*nagC*	This study
His-GlmS	pTac-*glmS*	This study
His-GlmM	pTac-*glmM*	This study
His-GlmU	pTac-*glmU*	This study
His-GlmR	pTac-*glmR*	This study
His-GNA1	pTac- *Saccharomyces cerevisiae* GNA1	This study
His-NagC R25A	pTac-*nagC* R25A	This study
His-NagC R35A	pTac-*nagC* R35A	This study
His-NagC R54A	pTac-*nagC* R54A	This study
His-NagC R59A	pTac-*nagC* R59A	This study
His-GlmR R110A	pTac-*glmR* R110A	This study
His-GlmR R212A	pTac-*glmR* R212A	This study
His-NagC quadruple R/K	pTac-*nagC* R25K R35K R54K R59K	This study
**Transcriptional fusions**		
*nagB*:*:gfp*	pHG156a- *nagB*::*gfp*	This study
*glmU*::*gfp*	pHG156a- *glmU*::*gfp*	This study
*glmR*::*gfp*	pHG156a- *glmR*::*gfp*	This study

*pTac is a pET28a derivative in which the T7 promoter was replaced with the Tac promoter.

**Table 2:** Strains used in this study.

Strain	Source
*Salmonella Typhimurium* ATCC 14028	^39^
*S. Typhimurium* Δ*sseK1*	^39^
*S. Typhimurium* Δ*sseK2*	^39^
*S. Typhimurium* Δ*sseK3*	^39^
*S. Typhimurium* Δ*sseK1*Δ*sseK2*	^39^
*S. Typhimurium* Δ*sseK1*Δ*sseK3*	^39^
*S. Typhimurium* Δ*sseK2*Δ*sseK3*	^39^
*S. Typhimurium* Δ*sseK1*Δ*sseK2*Δ*sseK3*	^39^
*E. coli* BL21(DE3) × pTac *nag*C (*S. Typhimurium*)	This study
*E. coli* BL21(DE3) × pTac *nagC* R25A	This study
*E. coli* BL21(DE3) × pTac *nagC* R35A	This study
*E. coli* BL21(DE3) × pTac *nagC* R54A	This study
*E. coli* BL21(DE3) × pTac *nagC* R59A	This study
*E. coli* BL21(DE3) × pTac-*nagC* R25K R35K R54K R59K	This study
*E. coli* BL21(DE3) × pTac *glmR* (*S. Typhimurium*)	This study
*E. coli* BL21(DE3) × pTac *glmR* R110A	This study
*E. coli* BL21(DE3) × pTac *glmR* R212A	This study
*E. coli* BL21(DE3) × pTac *glmU* (*S. Typhimurium*)	This study
*E. coli* BL21(DE3) × pTac *glmM* (*S. Typhimurium*)	This study
*E. coli* BL21(DE3) × pTac *glmS* (*S. Typhimurium*)	This study
*E. coli* BL21(DE3) × pTac *S. cerevisiae GNA-1*	This study
*S. Typhimurium* × pTac *glmU*	This study
*S. Typhimurium* × pTac *glmS*	This study
*S. Typhimurium* × pTac *glmM*	This study
*S. Typhimurium* × pTac *nagC*	This study
*S. Typhimurium* Δ*sseK1* × pTac *nagC*	This study
*S. Typhimurium* Δ*sseK2* × pTac *nagC*	This study
*S. Typhimurium* Δ*sseK3* × pTac *nagC*	This study
*S. Typhimurium* Δ*sseK1*Δ*sseK2* × pTac *nagC*	This study
*S. Typhimurium* Δ*sseK1*Δ*sseK3* × pTac *nagC*	This study
*S. Typhimurium* Δ*sseK2*Δ*sseK3* × pTac *nagC*	This study
*S. Typhimurium* Δ*sseK1*Δ*sseK2*Δ*sseK3* × pTac *nagC*	This study
*S. Typhimurium* × pTac *nagC* R25A	This study
*S. Typhimurium* × pTac *nagC* R35A	This study
*S. Typhimurium* × pTac *nagC* R54A	This study
*S. Typhimurium* × pTac *nagC* R59A	This study
*S. Typhimurium* × pTac *glmR*	This study
*S. Typhimurium* Δ*sseK1* × pTac *glmR*	This study
*S. Typhimurium* Δ*sseK2* × pTac *glmR*	This study
*S. Typhimurium* Δ*sseK3* × pTac *glmR*	This study
*S. Typhimurium* Δ*sseK1*Δ*sseK2* × pTac *glmR*	This study
*S. Typhimurium* Δ*sseK1*Δ*sseK3* × pTac *glmR*	This study
*S. Typhimurium* Δ*sseK2*Δ*sseK3* × pTac *glmR*	This study
*S. Typhimurium* Δ*sseK1*Δ*sseK2*Δ*sseK3* × pTac *glmR*	This study
*S. Typhimurium* × pTac *glmR R110A*	This study
*S. Typhimurium* × pTac *glmR R212A*	This study
*S. Typhimurium* × p*nagB promoter*::*gfp*	This study
*S. Typhimurium* × p*glmU promoter*::*gfp*	This study
*S. Typhimurium* × p*glmR promoter*::*gfp*	This study
*S. Typhimurium* Δ*sseK1 *× p*nagB promoter*::*gfp*	This study
*S. Typhimurium* Δ*sseK1 *× p*glmU promoter*::*gfp*	This study
*S. Typhimurium* Δ*sseK1 *× p*glmR promoter*::*gfp*	This study
*S. Typhimurium* Δ*sseK1 *× p*nagB promoter*::*gfp* × *psseK1*	This study
*S. Typhimurium* Δ*sseK1 *× p*glmU promoter*::*gfp* × *psseK1*	This study
*S. Typhimurium* Δ*sseK1 *× p*glmR promoter*::*gfp* × *psseK1*	This study
*S. Typhimurium* Δ*sseK1 *× p*nagB promoter*::*gfp* × *pHEN*	This study
*S. Typhimurium* Δ*sseK1 *× p*glmU promoter*::*gfp* × *pHEN*	This study
*S. Typhimurium* Δ*sseK1 *× p*glmR promoter*::*gfp* × *pHEN*	This study

### In vitro glycosylation assays

Assays were performed as described previously^12^. Briefly, 200 nM of SseK1 was incubated with 1 μM of either wild type or mutant forms of NagC or GlmR in buffer containing 50 mM Tris–HCl pH 7.4, 1 mM UDP-GlcNAc, 10 mM MnCl_2_, and 1 mM DTT. After 2 h incubation at room temperature, samples were subjected to western blotting using an anti-R-GlcNAc monoclonal antibody (Abcam).

### Digest of gel-separated proteins

SDS-PAGE separated affinity-purified proteins were fixed and visualized with Coomassie staining. Bands of interest were excised and then destained of Coomassie with 50 mM NH_4_HCO_3_, 50% ethanol for 20 min at room temperature with shaking at 750 rpm. Destained samples were then dehydrated with 100% ethanol, dried by vacuum-centrifugation for 20 min and then rehydrated in 50 mM NH_4_HCO_3_, 10 mM DTT. Samples were reduced for 1 h at 56 °C with shaking. Following reduction, the reduction buffer was removed, and the gel bands were washed twice in 100% ethanol for 10 min to remove residual DTT. Dehydrated gel bands were then rehydrated with 55 mM iodoacetamide in 50 mM NH_4_HCO_3_ and allowed to alkylate in the dark for 45 min at room temperature. The alkylation buffer was removed and gel samples washed with 50 mM NH_4_HCO_3_, followed by two rounds of 100% ethanol and vacuum dried. Alkylated samples were then rehydrated with either 20 ng/µl of trypsin (Promega) for NagC or 20 ng/ul of Glu-C (Promega) for GlmR in 40 mM NH_4_HCO_3_ at 4 °C for 1 h. Excess protease was removed, gel pieces were covered in 40 mM NH_4_HCO_3_ and incubated overnight at 37 °C. Peptides were collected, desalted using homemade R3/C18 stage tips as previously described^42^ before analysis by LC–MS.

### Reverse phase LC–MS/MS

Peptides were resuspended in Buffer A* (2% MeCN, 0.1% TFA) and separated using a two-column chromatography set on a Dionex Ultimate 3000 UHPLC (Thermo Fisher Scientific). Samples were first concentrated on a PepMap100 C18 20 mm × 75 μm trap at 5 μl/min for 5 min with Buffer A (0.1% formic acid, 2% DMSO) and then separated on a PepMap C18 500 mm × 75 μm analytical column (Thermo Fisher Scientific). Separated peptide were infused into a Q-Exactive plus Mass Spectrometer (Thermo Fisher Scientific) at 300 nl/minute for 119 min by altering the buffer composition from 2% Buffer B (0.1% formic acid, 77.9% acetonitrile, 2% DMSO) to 28% B over 90 min, then from 28% B to 4% B over 10 min, then from 40% B to 80% B over 5 min. The composition was held at 100% B for 5 min, and then dropped to 2% B over 1 min before being held at 2% B for another 9 min. The Q-Exactive plus Mass Spectrometer was operated in a data-dependent mode, acquiring one full precursor scan (resolution 70,000; 375–1800 m/z, AGC target of 1 × 10^6^) followed by 5 data-dependent HCD MS–MS events (using three collision energies of 28, 32, and 38; resolution 35 k AGC target of 2 × 10^5^ with a maximum injection time of 110 ms).

### Mass spectrometry data analysis

Identification of Arg-glycosylation events was accomplished using MaxQuant (v1.6.17.0)^43^. The predicted amino acid sequences for GlmR and NagC were combined into a database with the *Salmonella typhimurium* SL1344 proteome (Uniprot accession: UP000008962) and searched, allowing carbamidomethylation of cysteine set as a fixed modification and the variable modifications of oxidation of methionine and Arg-GlcNAcylation (H_13_C_8_NO_5_; 203.0793 Da to Arginine). Searches were performed with either Trypsin or GluC cleavage specificity depending on the protease used for digestion, allowing 2 miscleavage events with a maximum false discovery rate (FDR) of 1.0% set for protein and peptide identifications. The resulting modified peptide output was processed within the Perseus (v1.4.0.6)^44^ analysis environment to remove reverse matches and common protein contaminants. The mass spectrometry proteomics data have been deposited to the ProteomeXchange Consortium via the PRIDE^45^ partner repository with the dataset identifier PXD030710.

### GFP reporter assay

A low-copy number plasmid (pHG165) carrying *nagB*, *glmU*, or *glmR* promoter transcriptional fusions to *gfp* was electroporated into *Salmonella*. Two hundred µl of M9 minimal medium supplemented with either 0.2% glucose or 0.2% GlcNAc was used to grow the transformed bacteria in 96 well plates. GFP expression levels were measured after 8 h of growth and GFP data were presented as an average of RFU (relative fluorescence units)/OD_600_ ratio.

### EMSAs

Two nmoles of 5’ Alexa-fluor labeled DNA corresponding to *nagB*, *glmU*, or *glmR* promoters were incubated for 10 min at room temperature in the presence of either NagC or NagC-GlcNAc in a buffer containing 50 mM HEPES, 100 mM K glutamate (pH 8.0), and 0.5 mg/ml BSA. Samples (10 µl) samples were loaded on 0.5% agarose gels and subjected to electrophoresis in 0.5X TBE buffer. DNA–protein complexes were visualized by using a Li-COR Odyssey. Dissociation constant estimates were calculated by fitting the EMSA data (% bound and unbound DNA) using non-linear regression in GraphPad Prism.

### GlmS activity assay

GlmS activity assays were performed as previously described^26^. Briefly, reactions were performed in a buffer containing 50 mM Tris–HCl pH 7.4, 1 mM EDTA, 2 mM F6P, 2 mM L-Gln, 0.5 mM Ac-CoA, 0.5 mM DTNB, 10 µg GNA-1, and 1 µM GlmS, in the presence of absence of 1 µM native or Arg-glycosylated GlmR. GlmS activity was measured for 2 h at 37 °C by monitoring CoA production at 412 nm.

### Quantification of UDP-GlcNAc

*Salmonella* strains were grown overnight in M9 medium and cell lysates were incubated for 2 h at room temperature with 100 mM SseK1 to hydrolyze UDP-GlcNAc. The released UDP was quantified using a UDP detection reagent (Promega) that converts UDP into ATP to generate light in a luciferase reaction.

### Statistical analyses

Fluorescence and luminescence data were analyzed statistically using Dunn’s multiple comparisons. EMSA and enzyme assay data were analyzed statistically using Kruskal–Wallis tests. *p*-value < 0.05 were considered significant.
